# Diversification, selective sweep, and body size in the invasive Palearctic alfalfa weevil infected with *Wolbachia*

**DOI:** 10.1038/s41598-021-88770-y

**Published:** 2021-05-06

**Authors:** Midori Tuda, Shun-ichiro Iwase, Khadim Kébé, Julien Haran, Jiri Skuhrovec, Ehsan Sanaei, Naomichi Tsuji, Attila Podlussány, Ottó Merkl, Ahmed H. El-Heneidy, Katsura Morimoto

**Affiliations:** 1 Institute of Biological Control, Faculty of Agriculture, Kyushu University, Fukuoka, 819-0395 Japan; 2 Laboratory of Insect Natural Enemies, Department of Bioresource Sciences, Faculty of Agriculture, Kyushu University, Fukuoka, Japan; 3 GRBA-BE, LE3PI Laboratory, Department of Chemical Engineering and Applied Biology, Polytechnic Higher School of Dakar, Dakar, Senegal; 4 CBGP, Cirad, Montpellier SupAgro, INRA, IRD, Univ. Montpellier, Montpellier, France; 5 Group Function of Invertebrate and Plant Biodiversity in Agro-Ecosystems, Crop Research Institute, Drnovska, Praha, Czech Republic; 6 School of Biological Sciences, University of Queensland, Brisbane, Australia; 7 Entomological Laboratory, Graduate School of Bioresource and Bioenvironmental Sciences, Kyushu University, Fukuoka, Japan; 8 Hungarian Natural History Museum, Budapest, Hungary; 9 Department of Biological Control, Plant Protection Research Institute, Agricultural Research Center, Giza, Egypt; 10 Nata-Danchi, Fukuoka, Japan; 11 Present Address: Research Institute of Environment, Agriculture and Fisheries, Osaka Prefecture, Japan

**Keywords:** Phylogenetics, Population genetics, Bacteria, Evolution, Microbiology, Zoology, Ecology

## Abstract

The alfalfa weevil *Hypera postica*, native to the Western Palearctic, is an invasive legume pest with two divergent mitochondrial clades in its invading regions, the Western clade and the Eastern/Egyptian clade. However, knowledge regarding the native populations is limited. The Western clade is infected with the endosymbiotic bacteria *Wolbachia* that cause cytoplasmic incompatibility in host weevils. Our aim was to elucidate the spatial genetic structure of this insect and the effect of *Wolbachia* on its population diversity. We analyzed two mitochondrial and two nuclear genes of the weevil from its native ranges. The Western clade was distributed in western/central Europe, whereas the Eastern/Egyptian clade was distributed from the Mediterranean basin to central Asia. Intermediate mitotypes were found from the Balkans to central Asia. Most Western clade individuals in western Europe were infected with an identical *Wolbachia* strain. Mitochondrial genetic diversity of the infected individuals was minimal. The infected clades demonstrated a higher nonsynonymous/synonymous substitution rate ratio than the uninfected clades, suggesting a higher fixation of nonsynonymous mutations due to a selective sweep by *Wolbachia*. Trans-Mediterranean and within-European dispersal routes were supported. We suggest that the ancestral populations diversified by geographic isolation due to glaciations and that the diversity was reduced in the west by a recent *Wolbachia*-driven sweep(s). The intermediate clade exhibited a body size and host plant that differed from the other clades. Pros and cons of the possible use of infected-clade males to control uninfected populations are discussed.

## Introduction

Recent invasion events and routes of alien agricultural pests are of particular importance for the management and control of pests^1–6^. The knowledge of historical diversification and dispersal of agricultural pests in their native range provides insights to understand their natural and biological selective environments, including the role played by endosymbionts in pest emergence^7–9^.

The alfalfa weevil *Hypera postica* (Gyllenhal) (Coleoptera: Curculionidae: Hyperini), native to the Western Palearctic region, is a serious pest of alfalfa and other beneficial legumes in its invading territories, such as *Medicago*, *Vicia*, *Trifolium,* and *Astragalus*^10–12^ (Nearctic, Japan, Southeast Asia, and Oceania^13–15^). Invading populations in the USA comprise the Western (North American) type that invaded Utah in 1904^16^, the Egyptian type that invaded Arizona in 1939^17^, and the Eastern (North American) type that invaded Maryland in 1951^18^. These types are different in their ecological, behavioral, and defensive traits (pupation site and aggregation during aestivation^13^, defensive behavior^19^, and encapsulation of immature endoparasitoid^20–22^) but are morphologically indistinguishable. Allozyme and mitochondrial DNA markers distinguish the Western type from the Egyptian and Eastern types^22–24^, whereas the Egyptian and Eastern types are distinguishable only by a slight difference (1–2 SNPs in *tRNA*^*Ser*^) in a mitochondrial gene sequence^22,24,25^. The nuclear DNA polymorphism indicates that the three types share a gene pool, namely, a single species^25,26^. They also mate with each other to reproduce (but see later for incompatibility^27,28^).

*Wolbachia* (Alphaproteobacteria: Rickettsiales: Rickettsiaceae) are maternally (vertically) transmitted intracellular bacteria that infect approximately 40% of insects and other arthropods alongside nematodes^29^. These endosymbiotic bacteria can manipulate host reproduction via reproductive cells and the genetic mechanism of this manipulation has recently been uncovered^30^. Cytoplasmic incompatibility (CI), or postzygotic incompatibility between infected and uninfected gametes, is the most commonly observed phenotype of *Wolbachia*. Theoretically, bidirectional CI most strongly accelerates host speciation. Unidirectional CI, or postzygotic isolation of gametes between infected males and uninfected females, can also promote host speciation. While infection by *Wolbachia* is favored in females in populations with high *Wolbachia* prevalence, loss of *Wolbachia* can also occur through incomplete inheritance from mothers with low *Wolbachia* density^29^. The loss (or incomplete transmission) rate of *Wolbachia* in insect hosts is slightly higher than the gain rate^31^. Maternal transmission and unidirectional CI eventually reduce host mitochondrial diversity over generations. The selective sweep of mitochondria leads to a close association between the mitochondrial clade and *Wolbachia* infection^32–35^. Several studies have discovered that *Wolbachia* may also accelerate the fixation of nonsynonymous mutations in hosts^29,36–38^. Various positive fitness effects of endosymbionts on their hosts have been revealed, such as viral suppression and metabolic provisioning^39,40^. The effect of endosymbionts on maternal mitochondria may also influence coevolution between mitochondria and nuclear genomes^41,42^.

In *H. postica*, the Western clade is found to be infected by *Wolbachia* that induces unidirectional CI^27,43,44^. Several invading populations of the Western clade are free of *Wolbachia*, and a cross between uninfected Western males and Egyptian/Eastern females within these populations does produce viable offspring^28^. This reconfirms that these clades, while genetically distant, remain conspecific. The CI effect between infected Western males and uninfected Eastern females is almost perfect (only 0.1% of hybrid eggs hatch), while 29.5% of hybrid eggs between uninfected Eastern males and infected Western females hatch^27^.

The presence of diverged clades and *Wolbachia* infection history in Palearctic *H. postica* in its primary range is not known to date. Here, this study aims to explore the process of selection and diversification in *H. postica* in its native range by revealing and testing mitochondrial and nuclear genetic variation geographically and phylogenetically*.* We also aim to test if the endosymbiont *Wolbachia* affected evolution in host weevils. The benefits and risks of the Incompatible Insect Technique^45^ using infected clade males to control the uninfected clade populations are discussed.

## Results

### Haplotype networks and diversity

**Figure 1:**
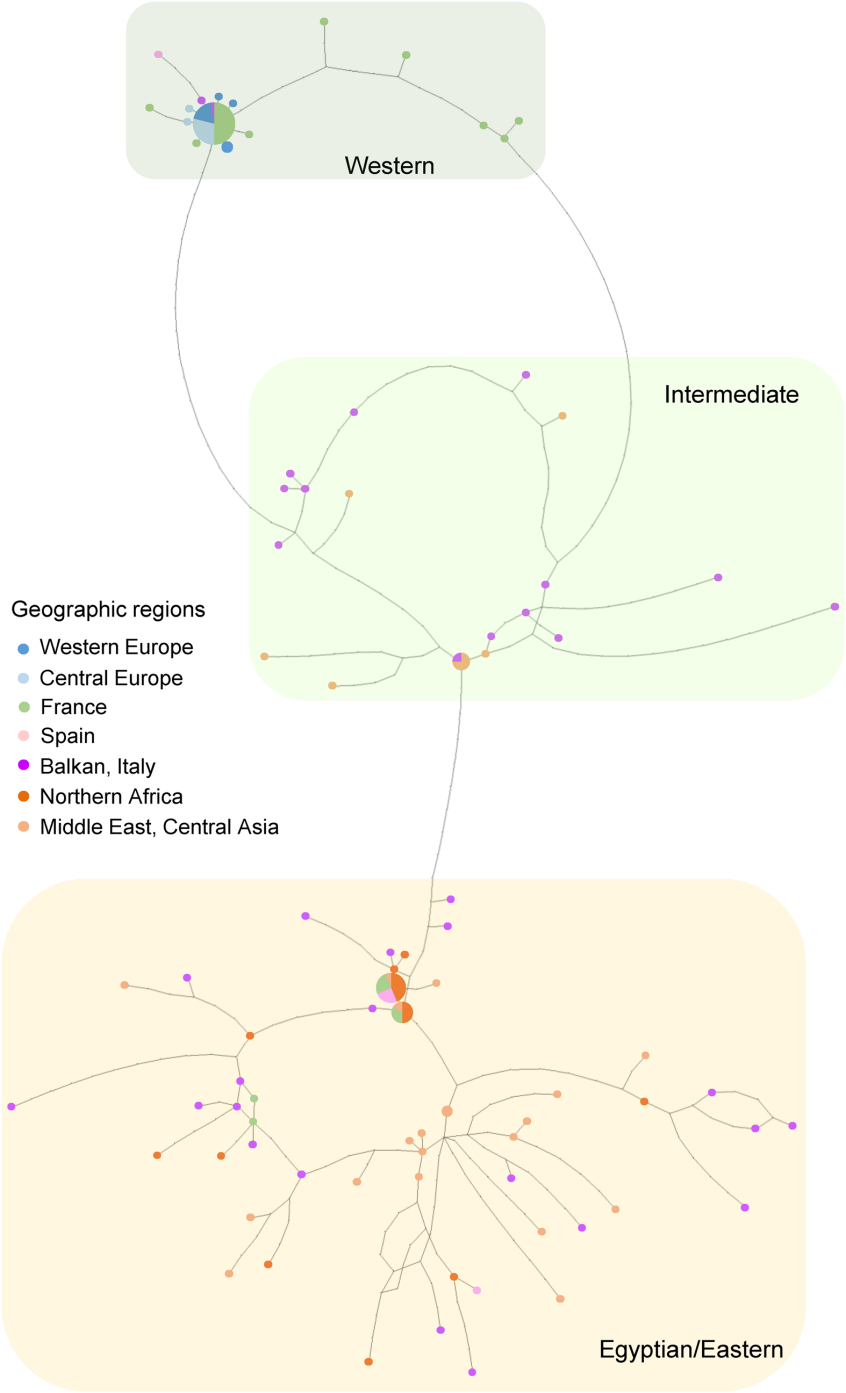
Statistical parsimony network of mitochondrial *COI*-*tRNA*^*Leu*^-*COII* and *Cyt b-tRNA*^*Ser*^*-ND1* of *Hypera postica* in its native range. Generated using TCS 1.21^80^.

**Figure 2:**
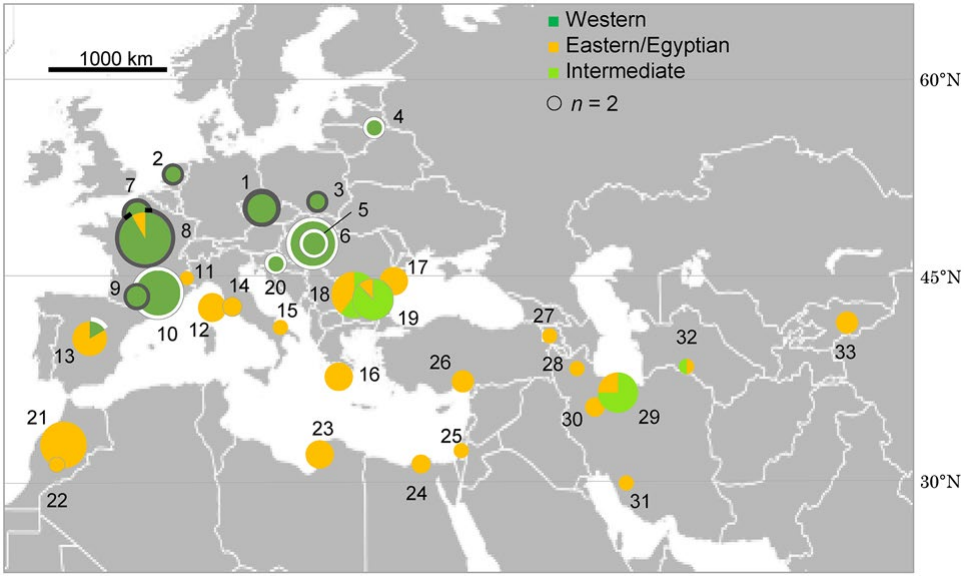
Geographic distribution of mitochondrial clades of *Hypera postica* in its native range. Pie chart sizes for clades are proportional to sample sizes. *Wolbachia* infection (dark gray ring: infected; white (partial) ring: uninfected) is only indicated for the Western clade individuals because none of the Egyptian/Eastern clade or the intermediate clade were infected. The background map was obtained from Fotolla, https://stock.adobe.com/jp/photos/.

**Figure 3:**
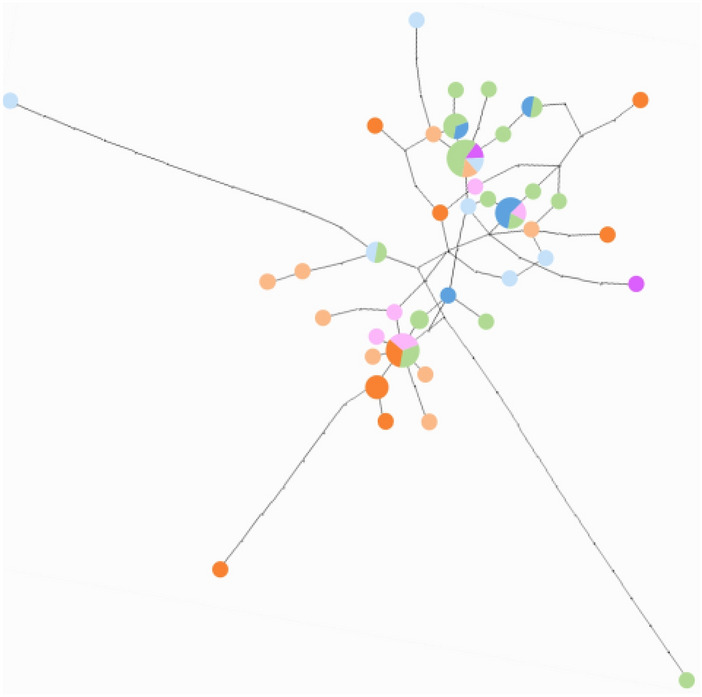
Statistical parsimony network of nuclear *28S* and *EF-1α.* See Fig. 1 for colors for regions. Generated using TCS 1.21^80^.

Sequenced segments were 2001 bp; 527 bp for *COI-tRNA*^*Leu*^*-COII*, 281 bp for *Cyt b-tRNA*^*Ser*^*-ND1* (*n* = 149), 801 bp for *28S* (*n* = 122) and 392 bp for *EF-1α* (*n* = 62) (Table 1). Despite our sizable effort, PCR failed for nuclear gene segments (especially *EF-1α*) for a part of the specimens. The mitotype network revealed two main clades alongside intermediate variants (Fig. 1). The first group corresponded to the Eastern/Egyptian clade (Fig. 1) and consisted of diverse mitotypes with multiple connections, which contributed to a high mitochondrial genetic diversity (Table 2). This clade was widely distributed from central Asia to the Mediterranean region (Fig. 2). Within this clade, populations from the Balkan peninsula displayed high mitochondrial and nuclear genetic diversity (Table 3). The second clade corresponded to the Western clade and exhibited substantially fewer mitotypes, one dominant mitotype and rarer, closely related mitotypes in a star-shape topology, which corresponds to low genetic diversity in both gene fragments (Fig. 1, Table 2). This clade was distributed in western and central Europe, north of the Alps and Pyrenees (Fig. 2). Within this clade, 50.0% of individuals and 50.0% of populations were infected with *Wolbachia* (Fig. 2)*.* The *Wolbachia*-infected populations demonstrated lower mitochondrial and nuclear genetic diversity than the uninfected populations (Table 2). Compared with the uninfected Eastern/Egyptian clade, the infected Western clade displayed 22 (*Cyt b-tRNA*^*Ser*^*-ND1*) to 82 (*COI-tRNA*^*Leu*^*-COII*) times lower mitochondrial genetic diversity (Table 2). The intermediate clade was distributed from the Balkans to central Asia (Fig. 2). The network for the nuclear fragments, *28S* and *EF-1α*, appeared incongruent with the mitochondrial network (Figs. 1, 3), but as in mitotype variation, there was a significant difference in nuclear haplotype variation between individuals belonging to the different mitochondrial clades (*EF-1α*, Table 2).

**Table 1:** Sample collection information for *Hypera postica.*CO: *COI*-*tRNA*^*Leu*^-*COII.* CB: *Cyt b-tRNA*^*Ser*^*-ND1.*

Number on the map	Code	Collection site	*n*	Year	Latitude, longitude	GenBank accession Mitochondrial genes	GenBank accession Nuclear genes
*Western Europe*							
1	Cz	Prague, Czech Republic	5	2012	50°05′16′'N,14°17′54′'E	KX372573 (CO), KX372620 (CB)	KX372667 (28S), MW392102 (EF1α)
2	Ne	Amsterdam, the Netherlands	2	2014	52°21′35′'N,4°57′00′'E	MW393903 (CO), MW393922 (CB)	MW383444 (28S), MW389094 (EF1α)
3	Po	Nida Basin, Poland	2	2012	50°24′N,20°38′E	MW393912 (CO), MW393931 (CB)	MW383460 (28S), MW389110 (EF1α)
4	Lit	Daugavpils, Latvia	2	2007	55°52′ N,26°27′E	MW393914 (CO), MW393933 (CB)	
*Central Europe*							
5	BuH	Budapest, Hungary	4	2012	47°28′41" N,19°01′00" E	MW393904 (CO), MW393923 (CB)	MW383445 (28S), MW389095 (EF1α)
6	AdH	Adyliget, Budapest, Hungary	9	2014	47°32′40" N,18°55′58" E	KX372574–75 (CO), KX372621–22 (CB)	MW383446 (28S), MW389096 (EF1α)
*France*							
7	ChF	Chaussy, France	4	2013	49°07′12"N, 1°42′02"E	MW393905 (CO), MW393924 (CB)	MW383448 (28S), MW389098 (EF1α)
8	OrF	Orléans, France	12	2013	47°53′59"N, 1°56′24"E	MW393906,16 (CO), MW393925,35 (CB)	MW383449,62 (28S), MW389099,112 (EF1α)
9	AuF	Auradé, France	3	2014	43°33′36"N, 1°03′01"E	KX372576 (CO), KX372623 (CB)	MW383447 (28S), MW389097 (EF1α)
10	ComF	Combaillaux, France	9	2016	43°40′12"N,3°46′47"E	MW393915 (CO), MW393934 (CB)	
11	AlpF	Saint-Paul-sur-Ubaye, France	2	2010	44°31′12"N, 6°45′02"E	KX372579 (CO), KX372626 (CB)	MW383450 (28S), MW389100 (EF1α)
12	CorF	Casamozza, Corse Island, France	5	2016	42°30′35"N,9°26′23"E	MW393910 (CO), MW393929 (CB)	MW383458 (28S), MW389108 (EF1α)
*Spain*							
13	Sp	La Cañada, Spain	6	2014	40°36′00"N,4°30′35"W	KX372577,82 (CO), KX372624,29 (CB)	MW383452,61 (28S), MW389102,11 (EF1α)
*Balkans and Italy*							
14	MaIt	Maremma, Toscana, Italy	3	2015	42°38′17"N, 11°07′29"E	MW393913 (CO), MW393932 (CB)	
15	PuIt	Puglia, Italy	2	2002	41°04′N,16°26′E	KX372599–600 (CO), KX372646,47 (CB)	MW383455 (28S), MW389105 (EF1α)
16	Gr	Sparti, Peloponnese, Greece	5	2005	36°51′ N, 22°39′ E	KX372586–87,614–16 (CO), KX372633–34,61–63 (CB)	
17	Ro	Crucea, Romania	5	2009	44°31′12" N, 28°11′59" E	KX372588,96–98,617 (CO), KX372635,43–45,64 (CB)	
18	KnBu	Knezha, Bulgaria	10	2016	43°28′48" N, 24°03′36" E	MW393917 (CO), MW393936 (CB)	
19	LoBu	Lozitsa, Bulgaria	8	2016	43°34′48"N, 25°00′01"E	MW393918 (CO), MW393937 (CB)	
20	Cr	Zagreb, Croatia	2	2006	45°50′35" N, 15°44′55" E	KX372578 (CO), KX372625 (CB)	
*Africa*							
21	faMo	Ouzoud falls, Morocco	10	2016	32°00′54"N,6°43′24"W	MW393909 (CO), MW393928 (CB)	MW383457 (28S), MW389107 (EF1α)
22	OuMo	Ouarzazate, Morocco	2	1994	30°56′N, 6°56′W	KX372589,618 (CO), KX372636,65 (CB)	
23	Li	Benghazi, Libya	5	1980	32°03′N,20°09′E	KX372603–04,13 (CO), KX372650–51,60 (CB)	
24	Eg	Sakha, Kafr El-Sheikh Governorate, Egypt	3	2013	31°05′13" N, 30°56′56" E	KX372580, MW393907 (CO), KX372627, MW393926 (CB)	MW383451 (28S), MW389101 (EF1α)
*Middle East and central Asia*							
25	Isr	Ga’ash, Israel	2	2014	32°13′48"N, 34°49′12"E	KX372590 (CO), KX372637 (CB)	MW383453 (28S), MW389103 (EF1α)
26	Tur	Catalan vill., Adana, Turkey	4	2002	37°15′01" N, 35°18′10" E	KX372581,601–02, MW393908 (CO), KX372628,48–49, MW393927 (CB)	
27	Ar	Metsamor, Armenia	2	2013	40°09′19"N, 44°07′30"E	KX372606 (CO), KX372653 (CB)	MW383456 (28S), MW389106 (EF1α)
28	AzIr	Azerbaijan, Iran	2	1999	37°56′ N, 47°23′ E	KX372584–85 (CO), KX372631–32 (CB)	
29	TaIr	Taleghan, Iran	8	2015	36°12′12"N,50°51′47"E	MW393919 (CO), MW393938 (CB)	
30	HaIr	Hamedan, Iran	3	2014	34°31′16"N,48°18′43"E	MW393911 (CO), MW393930 (CB)	MW383459 (28S), MW389109 (EF1α)
31	FaIr	Dasht-E-Arzhan, Zagros Mts., Fars Prov., Iran	2	2000	29°34′ N, 51°56′ E	KX372608–09 (CO), KX372655–56 (CB)	
32	Tm	Anau, Turkmenistan	2	1988	37°54′N,58°30′E	MW393920 (CO), MW393939 (CB)	
33	Ky	Jangy-Talap, Kyrgyzstan	4	2015	41°27′01"N, 75°01′12"E	KX372592 (CO), KX372639 (CB)	MW383454 (28S), MW389104 (EF1α)

**Table 2:** Mitochondrial and nuclear genetic diversity of the two clades (the Western and the Egyptian/Eastern) and the intermediate clades in *Hypera postica*. п: mean number of pairwise differences; nucleotide diversity (average over loci) (mean ± SD). The genetic distance was calculated based on pairwise differences. Numbers in parentheses are sample sizes (pooled number of individuals, number of populations). The same letters within each column (gene segment) indicate no significant difference between clades (*p* > 0.01). For population-wise mitochondrial and nuclear diversities and distances, see Supplementary Tables S1 and S2.

	Mitochondria						Nuclear					
	*COI*-*tRNA*^*Leu*^-*COII*			*Cyt b-tRNA*^*Ser*^*-ND1*			*28S*			*EF-1α*		
Clade (*Wolbachia* infection)	п	Nucleotide diversity		п	Nucleotide diversity		п	Nucleotide diversity		п	Nucleotide diversity	
Western (infected)	0.074 ± 0.150 (27, 6)	0.00014 ± 0.00031	a	0.148 ± 0.217 (27, 6)	0.00053 ± 0.00086	a	0.262 ± 0.299 (27, 6)	0.00033 ± 0.00042	a	3.134 ± 1.689 (22, 5)	0.00802 ± 0.00482	a
Western (uninfected)	3.481 ± 1.832 (27, 6)	0.00661 ± 0.00387		0.729 ± 0.560 (27, 6)	0.00260 ± 0.00222		1.057 ± 0.724 (25, 6)	0.00132 ± 0.00101		6.107 ± 3.251 (8, 3)	0.01554 ± 0.00942	
Egyptian/Eastern (uninfected)	6.057 ± 2.917 (74, 23)	0.01149 ± 0.00614	b	3.295 ± 1.714 (74, 23)	0.01173 ± 0.00676	b	1.052 ± 0.708 (60, 20)	0.00131 ± 0.00098	a	5.335 ± 2.646 (31, 12)	0.01334 ± 0.00736	b
Intermediate (uninfected)	10.300 ± 4.897 (21, 4)	0.01955 ± 0.01037	c	0.733 ± 0.567 (21, 4)	0.00261 ± 0.00225	c	2.111 ± 1.282 (10, 3)	0.00264 ± 0.00181	a	0 (1, 1)	0	ab
Among-clade variation (*df* = 2), *p*	72.53%, < 0.00001			87.60%, < 0.00001			3.34%, 0.0168			6.50%, 0.0544

**Table 3:** Mitochondrial and nuclear genetic diversity in *Hypera postica* based on geographic regions. п (mean number of pairwise differences); nucleotide diversity (average over loci) (mean ± SD). Bolded genetic diversity indices indicate the highest diversity for each gene segment. Numbers in parentheses are sample sizes (pooled number of individuals, number of sampled populations). See Table 1 for the country codes. For population-wise mitochondrial and nuclear diversities and distances, see Supplementary Tables S1 and S2.

Geographic region (country)	Mitochondria				Nuclear			
	*COI*-*tRNA*^*Leu*^-*COII*		*Cyt b-tRNA*^*Ser*^*-ND1*		*28S*		*EF-1α*	
	п	Nucleotide diversity	п	Nucleotide diversity	п	Nucleotide diversity	п	Nucleotide diversity
Western Europe (Cz, Ne, Lit, Po)	0.51 ± 0.46 (11, 4)	0.0010 ± 0.0010	0.18 ± 0.25 (11, 4)	0.0006 ± 0.0010	0.40 ± 0.40 (10, 4)	0.0005 ± 0.0006	3.07 ± 1.85 (6, 2)	0.0078 ± 0.0055
France (Fr)	9.92 ± 4.65 (35, 6)	0.0188 ± 0.0098	7.06 ± 3.40 (35, 6)	0.0251 ± 0.0134	0.58 ± 0.48 (35, 6)	0.0007 ± 0.0007	4.48 ± 2.30 (21, 6)	0.0112 ± 0.0064
Spain (Sp)	9.13 ± 4.90 (6, 1)	0.0173 ± 0.0107	**8.47** ± 4.57 (6, 1)	**0.0301** ± 0.0188	0.93 ± 0.74 (6, 1)	0.0012 ± 0.0011	2.67 ± 1.65 (6, 1)	0.0068 ± 0.0049
Central Europe (H)	0.00 ± 0.00 (13, 2)	0.0000 ± 0.0000	0.31 ± 0.34 (13, 2)	0.0011 ± 0.0014	0.73 ± 0.58 (12, 2)	0.0009 ± 0.0008	**6.10** ± 3.30 (7, 2)	**0.0155** ± 0.0096
Balkan and Italy (Gr, Bu, Ro, Cr, It)	**15.53** ± 7.10 (35, 7)	**0.0295** ± 0.0150	5.08 ± 2.53 (35, 7)	0.0181 ± 0.0100	**1.32** ± 0.85 (23, 7)	**0.0016** ± 0.0012	**6.00** ± 4.58 (2, 2)	**0.0153** ± 0.0166
Africa (Mo, Lib, Eg)	4.23 ± 2.19 (20, 4)	0.0080 ± 0.0046	2.58 ± 1.44 (20, 4)	0.0092 ± 0.0057	1.00 ± 0.71 (16, 4)	0.0012 ± 0.0010	4.62 ± 2.42 (13, 3)	0.0117 ± 0.0069
Middle East and central Asia (Isr, Ir, Ar, Tur, Tm, Ky)	10.23 ± 4.81 (29, 9)	0.0194 ± 0.0102	3.08 ± 1.65 (29, 9)	0.0110 ± 0.0065	1.08 ± 0.74 (20, 8)	0.0013 ± 0.0010	5.05 ± 2.79 (7, 4)	0.0129 ± 0.0082
Among geographic region variation, *p*	36.6%, < 0.00001		63.0%, < 0.00001		13.0%, < 0.00001		9.2%, 0.00198

### *Wolbachia* infection

**Figure 4:**
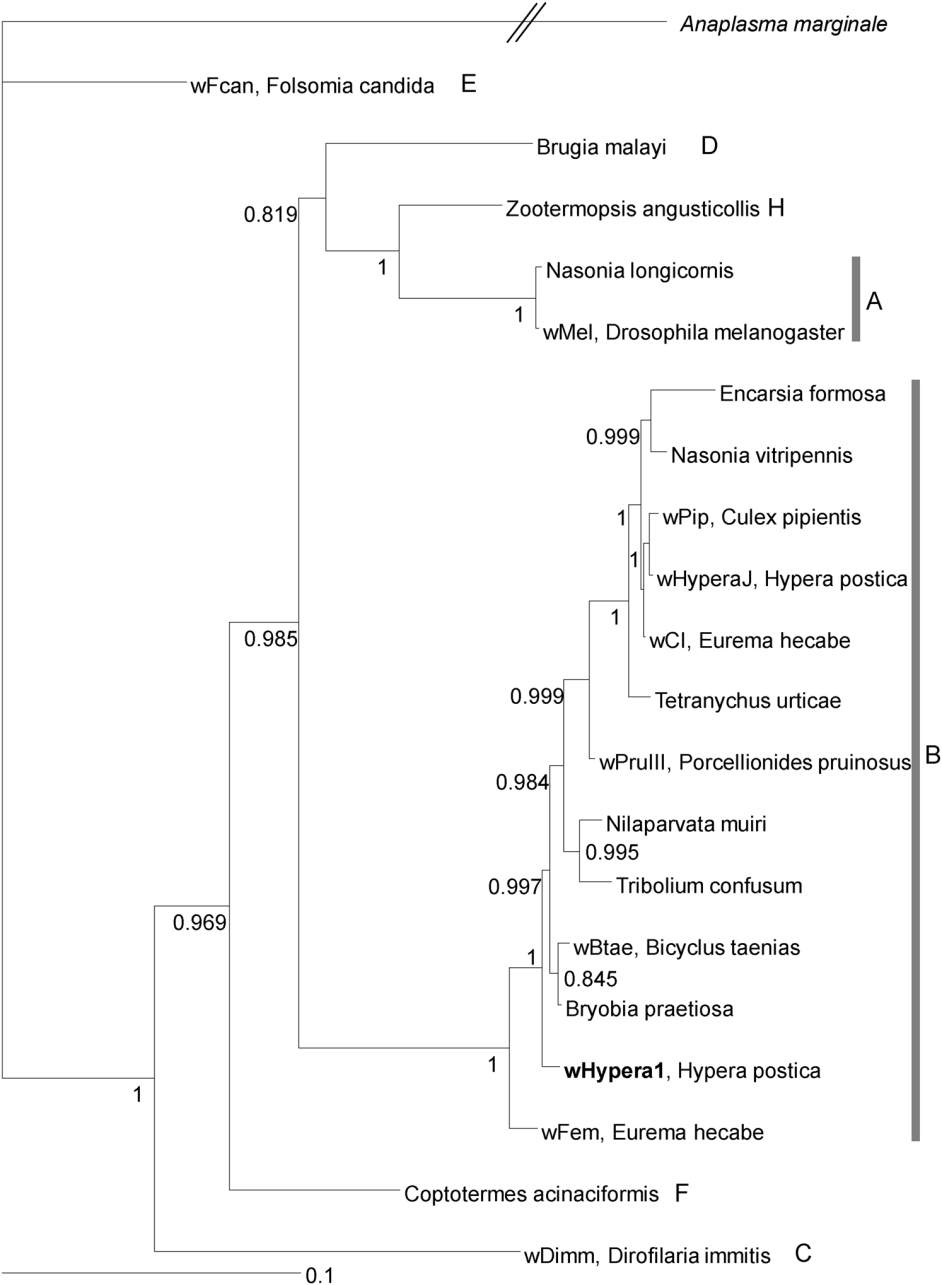
Bayesian consensus tree of *Wolbachia* strain based on *ftsZ*, *coxA,* and *hcpA*. Strain codes, if available, followed by host species and *Wolbachia* supergroups are shown. The *Wolbachia* strain, wHypera1, infecting *Hypera postica* in its native range, is shown in bold. Bayesian support values (posterior probabilities > 0.7) are shown near nodes. The outgroup is *Anaplasma marginale* (Alphaproteobacteria: Rickettsiales: Anaplasmataceae)*.* Generated using MrBayes 3.2.6^73^. Host strains, *Wolbachia* isolates and GenBank accession numbers are listed in Supplementary Table S3.

The Western clade individuals in northern France, the Netherlands, the Czech Republic, and Poland were infected with *Wolbachia* (Fig. 2), and all of them had identical *ftsZ* (699 bp), *coxA* (432 bp), and *hcpA* (463 bp) sequences as reported previously^46^, which corresponds to the prevailing strain wHypera1^47^. In contrast, Western clade individuals found in coastal southern France, Spain, Latvia, Hungary, and Croatia were uninfected (Fig. 2). Additionally, we confirmed that *Wolbachia* was absent from all native populations studied in the Eastern/Egyptian clade. A reconstructed phylogenetic relationship confirms that wHypera1 belongs to Supergroup B (Fig. 4). The *Wolbachia* strain closest to wHypera1 to date is the one that infects the mite *Bryobia praetiosa* (Acari: Tetranychidae)^48^.

### Body size

Elytral length was significantly different among different clades (*χ*^2^_2_ = 10.96, *p* = 0.004) and between sexes (*χ*^2^_1_ = 7.52, *p* = 0.006) and marginally different between infected and uninfected individuals (*χ*^2^_1_ = 3.77, *p* = 0.052). The elytron was longer in the intermediate variants (3.83 ± 0.110 mm, mean ± SE, *n* = 21) than in the Egyptian/Eastern (3.48 ± 0.025 mm, *n* = 64, *p* = 0.003) and Western (3.50 ± 0.057 mm, *n* = 29, *p* = 0.041) clades (no difference between the Egyptian/Eastern and Western clades, *p* = 0.934), longer in females (3.62 ± 0.042 mm, *n* = 60) than in males (3.47 ± 0.044 mm, *n* = 52), and marginally longer in uninfected individuals (3.57 ± 0.032 mm, *n* = 102) than in infected individuals (3.36 ± 0.111 mm, *n* = 11).

### Selective neutrality test and positive selection test

For the Western clade, selective neutrality for mitochondrial segments was rejected by all indexes with minus values (*D*, D*, and F*), while for nuclear genes, selective neutrality was rejected by none of the indices (Table 4), suggesting recent sudden population growth after bottleneck event(s) in the mitochondrial lineage. For the Eastern/Egyptian clade, selective neutrality for both mitochondrial and nuclear segments was rejected by all indexes (Table 4), suggesting recent sudden population growth after bottleneck event(s) in the mitochondrial lineage and nuclear variants. For the intermediate clade, selective neutrality for mitochondrial segments was rejected by none of the indexes (Table 4). Selective neutrality for nuclear segments was untested because of the insufficient sample size.

**Table 4:** Selective neutrality test results on mitochondrial and nuclear segments. *D*: Tajima's *D*; D* and F*: Fu and Li's D* and F*.

Clade	Mitochondria				Nuclear			
	*D* (*p*)	D* (*p*)	F* (*p*)	*n*	*D* (*p*)	D* (*p*)	F* (*p*)	*n*
Western	− 2.389 (0)	− 3.371 (0.0015)	− 3.302 (0.0010	23	− 0.822 (0.152)	− 0.547 (0.267)	− 0.606 (0.240)	15
Egyptian/Eastern	− 1.510 (0.039)	− 2.872 (0.0175)	− 2.673 (0.018)	39	− 1.491 (0.0255)	− 2.007 (0.0125)	− 1.959 (0.0165)	19
Intermediate	0.695 (0.763)	0.566 (0.707)	0.596 (0.719)	6				

A model with different *ω* (dN/dS) assigned for infected and uninfected clades improved the model fit most, compared to models with different *ω* for two or three different clades, although the improvement was nonsignificant (Table 5). The *ω* for infected clade was three times higher than the *ω* for uninfected clade even though both were < 1 (Table 5).

**Table 5:** Test of positive selection on the *Hypera postica* phylogeny. Root: *Brachypera zoilus* and *H. miles*. W: Western. E: Egyptian/Eastern. Inf: infected by *Wolbachia*. Uninf: uninfected by *Wolbachia*. ΔlnL: difference in log likelihood (lnL) of each model from the model with a same single *ω* for W, intermediate, and E clades (i.e., the ‘Root and W/intermediate/E’ model as a reference model). Δdf: difference of each model in degree of freedom (df) from the model with a same single *ω* for W, intermediate, and E clades. The – symbol: the same *ω* value as the one on the left.

Model (number of *ω*)	Root	E	Intermediate	W	InfW	2ΔlnL	Δdf	*p*
Root and W/intermediate/E (2)	0.0103	0.0578	–	–	–	0	0	1.000
Root, W and intermediate/E (3)	0.0103	0.0575	–	0.0590	–	0	1	1.000
Root, W/intermediate and E (3)	0.0104	0.0541	0.0640	–	–	1.50	1	0.221
Root, InfW and UninfW/intermediate/E (3)	0.0103	0.0571	–	–	0.1709	1.80	1	0.180
Root, InfW, UninfW and intermediate/E (4)	0.0103	0.0575	–	0.0554	0.1709	1.82	2	0.403
Root, InfW, UninfW/intermediate and E (4)	0.0104	0.0541	0.0620	–	0.1709	0.72	2	0.698

### Geographic history

#### Isolation by distance

Isolation by distance (IBD) was supported for all populations (*p* = 0.003, number of pairwise comparisons *n* = 528) and for the Eastern/Egyptian clade excluding populations with intermediate mitotypes (*p* = 0.050, *n* = 153), but not for the Western clade (*p* = 0.102, *n* = 55). When the populations with intermediate mitotypes were included, the IBD within the Eastern/Egyptian clade was not supported (*p* = 0.215, *n* = 231).

#### Phylogeography

**Figure 5:**
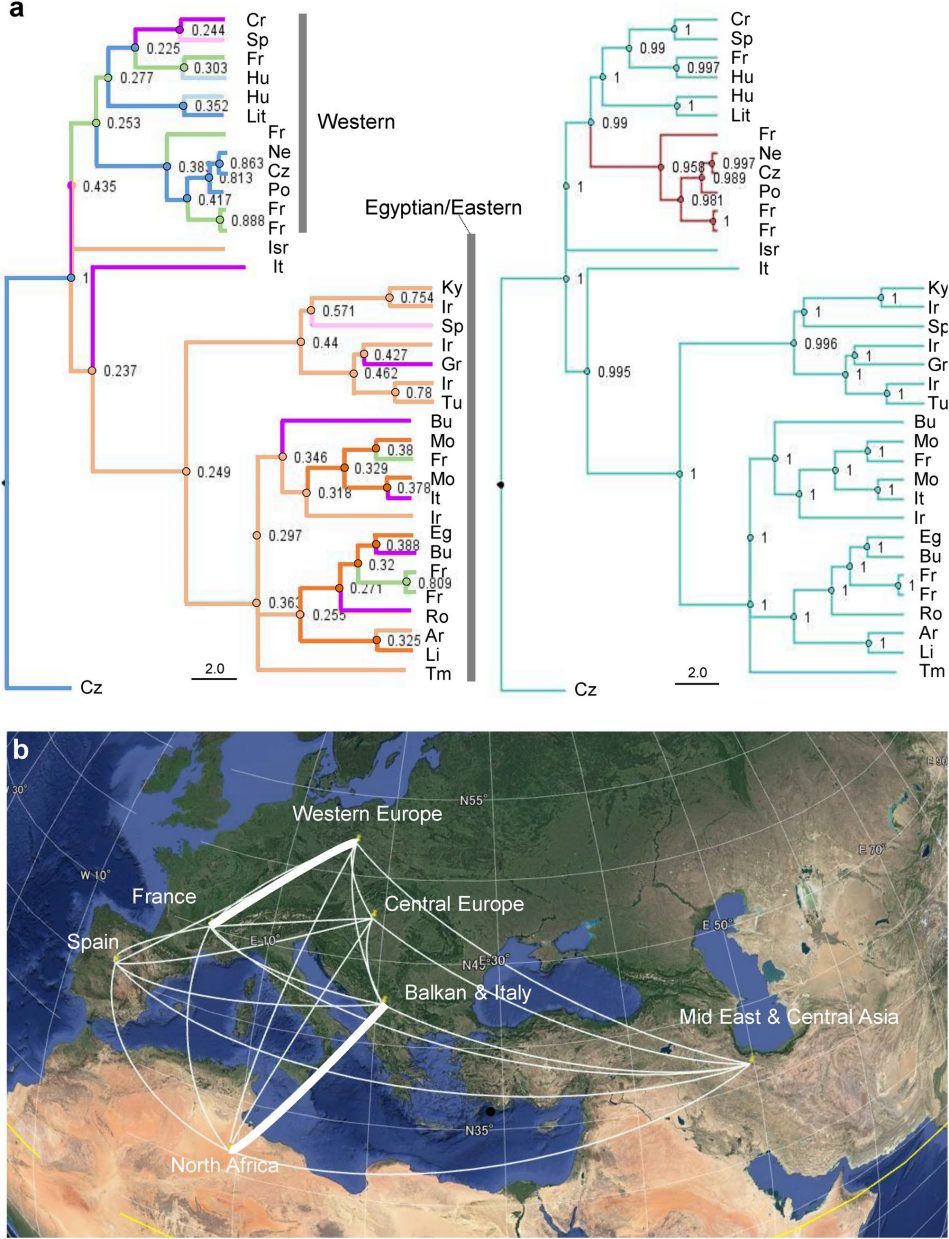
(**a**) The maximum clade credibility (MCC) tree of *Hypera postica* resulting from a discrete Bayesian phylogeographic analysis, based on one sample per clade per country. The outgroup is *H. miles*. The nodes and branches are colored according to the most probable region (left panel; see Fig. 1 for colors indicating geographic regions and Table 1 for the country codes) and *Wolbachia* infection state (right panel; red line: infected). The numbers on the nodes indicate posterior probabilities of the most likely ancestral states [geographic region (left panel) or *Wolbachia* infection state (right panel)]. Generated using BEAST 1.10.4^90^ and visualized using FigTree 1.4.4 (http://tree.bio.ed.ac.uk/software/figtree/). (**b**) Map of colonization routes of *H. postica*. Thick lines: supported by BF (Bayes factor) > 7.0. Generated using SpreaD3 v0.9.7.1rc^92^ and visualized using Google Earth 7.3.0 (https://www.google.com/earth/).

Based on available samples, the Balkan/Italian peninsulas and the Middle East are the most likely area of the origin of *H. postica*, from which the Western clade diversified via France (Fig. 5a). France is the likely area where the ancestral population was first infected with *Wolbachia* (Fig. 5a, right). We found two connections between regions that were highly supported with BF > 3: France and western Europe (BF = 7.98) and Balkan/Italy and North Africa (BF = 7.90) (BSSVS analysis, Fig. 5b).

## Discussion

This study revealed the large-scale geographic distribution and genetic diversity of *H. postica* in its native range. Intermediate mitotypes with larger body sizes were found from the Balkans to central Asia. The observed reduced diversity within the Western clade is likely due to a high percentage of *Wolbachia* infection within this clade, which is known in other species. We also identified a higher substitution rate of nonsynonymous mutations, suggesting promoted (fixation of) nonsynonymous mutations in the infected Western clade. In the Western clade, recent sudden population growth after a bottleneck was suggested only for mitochondrial genes and not for nuclear genes, supporting a recent selective sweep on mitochondria by *Wolbachia* infection.

Our results demonstrated a clear pattern of geographic distribution of the two divergent mitochondrial clades across the area of study, the Eastern/Egyptian and Western clades. The low genetic variation and star-like haplotype network within the Western clade is a signature of a recent demographic expansion from a few founders^49^. If the populations experienced ancient demographic bottlenecks, mitochondrial and nuclear genes are expected to have a concordant population structure. There was a weak concordance (Table 2), suggesting that these genes may have shared similar evolutionary trajectories. Two bottleneck events are likely; postglacial recolonization (see next section) and a recent mitochondrial sweep by *Wolbachia*. The former may also serve as a major driver of IBD that was supported for overall geographic populations (two clades distributed separately in the north and south with intermediate mitotypes in between). The latter likely accelerates the fixation of nonsynonymous mutations in the Western clade^33,35,50–53^.

The asymmetric inheritance of maternal mitochondria of an infected host caused by unidirectional CI-inducing *Wolbachia* can eventually lead to a sweep, which likely explains the low mitochondrial genetic variation among infected individuals. The infected clade demonstrated accelerating nonsynonymous mutations or fixation. This result is consistent with a general trend of *Wolbachia* infected insect groups^38^, suggesting fixation of nonsynonymous mutations in mitochondria promoted by its small effective population size under the CI-inducing *Wolbachia* infection. Furthermore, *Wolbachia* infection is advantageous for *H. postica* by enhancing resistance against its adult parasitoid *Microctonus aethiopoides*^22^.

In the southernmost populations of the Western clade or the geographic contact zone between the two clades, most individuals were uninfected or had lost *Wolbachia*. The imperfect maternal transmission was observed in the interclade crosses of *H. postica*^27^; fitness costs incurred by cytoplasmic incompatibility and stochasticity during the invasion process^45,54^ may lower the *Wolbachia* infection rate. Environmental causes (e.g., extreme temperatures) may also accelerate the endosymbiont loss^55,56^. Resulting uninfected *H. postica* populations (or with lowered *Wolbachia* density) must have regained reproductive compatibility between clades and enabled crosses between the diverged clades.

The intermediate variants exhibited a large body size. Larger genitalia of the males with these mitotypes may inhibit mating with the females of other clades and promote reproductive isolation. These mitotypes also were associated with an ecological niche that differed from the niche of other clades. Bulgaria populations used *Vicia cracca* as a host plant, whereas other populations used *Medicago* and *Trifolium*. *Vicia cracca* has high contents of cyanamide^57^ and canavanine^58^ that are toxic to insect herbivores^59,60^.

The genetic structure of most European biota has been strongly influenced by glacial oscillations of the Holocene^61,62^, and most temperate species exhibit northward post-glacial recolonization from glacial refugia located in southern Europe through central Asia during the last glacial maxima (southern genetic richness/northern purity^63^; in beetles^64^). In *H. postica*, we observed mtDNA differentiation for all clades and within the Eastern/Egyptian clade. Based on the estimated ancestral states in mitochondrial phylogeography and mitochondrial/nuclear genetic diversity, the Balkan and Italian peninsulas are a possible candidate for the origin of the Eastern/Egyptian clade and western Europe (France) for that of the Western clade. The primary center of genetic and species diversity of the main host *Medicago* is the Caucasus (north-western Iran and north-eastern Turkey)^65^, which may also consist of the area of origin of *H. postica*.

The recent dispersal routes that include the north Mediterranean were highly supported. Anthropogenic factors may allow occasional dispersal of *H. postica* to Europe and North Africa with alfalfa traded for livestock feed (by 2,600 years ago^66^). More recent international trade of alfalfa meal and pellets may continue to aid the weevil’s opportunistic long-distance dispersal; France, Spain, and Italy are the major alfalfa exporters among *H. postica*’s native ranges^67^.

### Conclusion

While geographic isolation assisted continental diversification of the weevil *H. postica*, recent *Wolbachia* infection reduced diversity in a mitochondrial clade in the host weevil in western Europe. *Wolbachia*-infected males could be used as a control agent for the Incompatible Insect Technique on uninfected populations, however, the risk of heterosis in interclade crosses following accidental cure of *Wolbachia* must be assessed before application.

## Methods

### Sampling

*Hypera postica* (*n* = 149) were obtained from 33 localities covering most of its native distribution range (Table 1). Adults were collected from cultivated and wild legume vegetation of *Medicago*^47^, *Trifolium* in Egypt, and *Vicia cracca* in Bulgaria, mostly during the latest decade. The samples were then stored in ethanol at 4 °C until DNA extraction*.*

### PCR and sequencing

DNA was extracted from all specimens using a DNeasy Blood & Tissue kit (Qiagen, Tokyo, Japan). We amplified and sequenced two mitochondrial fragments, *COI*-*tRNA*^*Leu*^-*COII* and *Cyt b-tRNA*^*Ser*^*-ND1* and two nuclear fragments, *28S* and *EF-1α.* The primers used were C1-J-2797^68^ and C2-N-3380^24^ for the *COI*-*tRNA*^*Leu*^-*COII* fragment, CB-J-11545 and N1-N-11841^68^ for *Cyt b-tRNA*^*Ser*^*-ND1*, 28S-01 and 28SR-01 for *28S*^69^, and ef415F (5′-AACCAGAGAACATGCTCTTCTCG-3′) and ef862R (5′-CTCAATTTTTTAAGTTTGTTCAATTTG-3′) (this study) for *EF-1α*. PCRs were performed using GoTaq Green Master Mix (Promega, Tokyo, Japan). Cycling conditions for *COI-tRNA*^*Leu*^*-COII* amplification consisted of preheating at 95 °C for 2 min, followed by 38 cycles of denaturation at 94 °C for 50 s, annealing at 50 °C for 1 min, and an extension at 60 °C for 1 min. Amplification conditions for *Cyt b-tRNA*^*Ser*^*-ND1* were identical, except that annealing was performed at 55 °C for 1 min. Cycling conditions for *28S* amplification were as follows; preheating as above, followed by 35 cycles of denaturation at 94 °C for 30 s, annealing at 52 °C for 40 s, and an extension at 70 °C for 1 min. Those for *EF-1α* consisted of preheating as above, followed by 38 cycles of denaturation at 94 °C for 30 s, annealing at 51 °C for 40 s, and an extension at 68 °C for 1 min. Sequencing was carried out using a BigDye Terminator v3.1 Cycle Sequencing kit (Life Technologies/Applied Biosystems, Foster City, CA, USA) on a 3730 DNA Analyzer (Applied Biosystems).

### *Wolbachia* infection and phylogeny

We used PCR to screen for possible *Wolbachia* infections. The *Wolbachia ftsZ* coding fragment was amplified using the primers fts-Z-f and fts-Z-r^70^. PCRs were performed using preheating as above, followed by 32 cycles at 94 °C for 40 s, 55 °C for 45 s, and 70 °C for 1 min. As a positive control, we used *Wolbachia*-infected *Callosobruchus chinensis*^71^. Blurred and extremely weak signals compared with the positive control were considered uninfected, which differs from a previous study^47^. *Wolbachia*-positive *H. postica* were further subjected to PCR and sequencing of the genes, *coxA* and *hcpA*, in addition to *ftsZ* for multilocus sequence typing of *Wolbachia*^72^.

For the phylogenetic reconstruction of *Wolbachia*, we used sequences from representative supergroups of *Wolbachia* (nr/nt database, Supplementary Table S1) with sequences of *Anaplasma marginale* as an outgroup. We used the GTR model, which was selected as the best fit model of nucleotide substitution by MrBayes3.2.6^73^, based on the AICc, using MrAIC.pl 1.3.1^74^. The three gene segments were partitioned. Markov chain Monte Carlo (MCMC) simulations were performed for one million generations, with sampling conducted every 1,000 generations. The convergence of independent parallel runs was checked using Tracer 1.6^75^, and the first 25% of trees were discarded as burn-in.

### Body size

After collecting specimens, the right elytron lengths of the samples were measured to the precision of 0.01 mm with a microscope (VH-5500, Keyence, Osaka, Japan). The sex of the samples was determined by both external and genital morphology. The effect of sex, clades (Egyptian/Eastern, Western, and intermediate), and *Wolbachia* infection on elytral lengths were tested by nonparametric Wilcoxon/Kruskal-Wallis signed rank tests. Posthoc multiple comparison was performed on the significant factor using the Steel-Dwass test. JMP 14.2.0 was used for statistical analyses.

### Selective neutrality test and positive selection test

Selective neutrality was tested in each clade with Tajima^76^^′^’s *D*, Fu and Li^77^^′^’s D* and F*, using DNASP 6.12.03^78^. *P* values were derived by coalescent simulations with 2,000 replications. For the coalescent simulations for nuclear segments, an intermediate recombination rate was assumed. We used all mitochondrial (808 bp) or nuclear sequences (1,193 bp) of two individuals sampled per clade from each population to avoid sample size bias between populations.

The equal nonsynonymous/synonymous substitution rate ratio (dN/dS ratio, *ω*) between infected and uninfected clades and between the two major clades was tested with a phylogenetic analysis using the maximum likelihood method (likelihood ratio test) employed by the codeml program in PAML 4.9i^79^. The models with three or more different *ω* for each branch were compared with a reference (basal) model with two different *ω* (one for the root and the other for both the Western and Egyptian/Eastern clades). We concatenated all the open reading frames (protein-coding fragments) and removed potential stop codons (leading to 215 codons) of the mitochondrial sequences of two individuals randomly sampled per clade from each population to avoid sample size bias between populations. Codons for invertebrate mitochondria were used.

### Haplotype networks and diversity

All mitochondrial sequences were assembled using Sequencher 5.0 (Gene Codes Corp, Ann Arbor, MI, USA), and we checked for the presence of pseudogenes using commonly employed methods^80,81^. Statistical parsimony networks were reconstructed based on mitochondrial and nuclear fragments using TCS 1.21^82^, in which we allowed connections between haplotypes of 20 steps for mitochondrial genes and 95% for the nuclear genes, to elucidate the maximal divergence observed among haplotypes. Nucleotide diversity (average over loci) and п (mean number of pairwise differences)^83^ were estimated for each geographic area, using Arlequin 3.5.2.2^84^.

### Geographic history

#### Isolation by distance

We assessed spatial mitochondrial differentiation by testing for isolation by distance (IBD)^85^. With a sweep (e.g., by *Wolbachia*) followed by rapid spread or frequent anthropogenic long-distance dispersal events, the IBD correlation is predicted to be weak at most. We tested if, as predicted by IBD, pairwise geographic distances and pairwise genetic differences were positively correlated using a one-tailed Mantel test^86^ based on 2,000 permutations with the ISOLDE program implemented in Genepop 4.2^87^. For pairwise genetic differences, we employed corrected average pairwise differences between populations X and Y, [π_XY_ − (π_X_ + π_Y_)/2]^88^ and their *p* values were derived using Arlequin.

#### Phylogeography

We estimated the historical dispersal patterns of *H. postica*, using a Bayesian discrete phylogeographic approach^89^ with a Bayesian skyride framework implemented in the software package BEAST 1.10.4^90^. We used two mitochondrial segments (808 bp). To avoid sample size bias, we selected only one individual per clade from a given locality but excluded intermediate mitotypes, which reduced the data set to 34 individuals, with *H. miles* as an outgroup. We used default settings, applied the same molecular evolution model as presented above, and used an uncorrelated relaxed clock model assuming lognormal rate distribution^91^. We assigned each sequence to one of the seven geographic regions, and the symmetric exchanges between the geographic regions throughout the entire phylogeny were modeled with the Bayesian stochastic search variable selection (BSSVS). MCMC runs were performed for 50 million generations, sampling one tree every 25,000 generations. After confirming the stationarity of parameter estimates using Tracer, the first 40% of trees were discarded as burn-in, and maximum clade credibility (MCC) tree was built using TreeAnnotatorv1.10.4. As each node in each MCMC sample is annotated with a geographic region and *Wolbachia* infection status, we assessed the certainty of the geographic reconstruction by looking at the distribution of node states across the MCMC using FigTree v1.4.4 (http://tree.bio.ed.ac.uk/software/figtree/). Bayes factor (BF) values for exchange rates between each pairwise regions were retrieved from the log file from the BSSVS analysis using SpreaD3 v0.9.7.1rc^92^.

## Supplementary Information

Supplementary Information.

## Data Availability

GenBank accessions KX372573–372592, 372596–372619 and MW 393902–393920 for *COI-tRNA*^*Leu*^*-COII*, KX372620–372639, 372643–372666 and MW393921–393939 for *Cyt b-tRNA*^*Ser*^*-ND1*, KX372667 and MW383443–383462 for *28S*, MW389094–389112 and 392102 for *EF-1α*, and MW389113–389118 for *ftsZ*, *coxA* and *hcpA.*

## References

[1] Bonizzoni M (2004). On the origins of medfly invasion and expansion in Australia. Mol. Ecol..

[2] Tuda M, Kagoshima K, Toquenaga Y, Arnqvist G (2014). Global genetic differentiation in a cosmopolitan pest of stored beans: Effects of geography, host-plant usage and anthropogenic factors. PLoS ONE.

[3] Karsten M, van Vuuren BJ, Addison P, Terblanche JS (2015). Deconstructing intercontinental invasion pathway hypotheses of the Mediterranean fruit fly (*Ceratitis capitata*) using a Bayesian inference approach: Are port interceptions and quarantine protocols successfully preventing new invasions?. Divers. Distrib..

[4] Rodriguero MS (2016). Out of the forest: past and present range expansion of a parthenogenetic weevil pest, or how to colonize the world successfully. Ecol. Evol..

[5] Kébé K (2017). Global phylogeography of the insect pest *Callosobruchus maculatus* (Coleoptera: Bruchinae) relates to the history of its main host *Vigna unguiculata*. J. Biogeogr..

[6] Lombaert E (2018). Colonization history of the western corn rootworm (*Diabrotica virgifera virgifera*) in North America: insights from random forest ABC using microsatellite data. Biol. Invasions.

[7] Tuda M, Ronn J, Buranapanichpan S, Wasano N, Arnqvist G (2006). Evolutionary diversification of the bean beetle genus *Callosobruchus* (Coleoptera: Bruchidae): Traits associated with stored-product pest status. Mol. Ecol..

[8] Wei SJ (2015). Population genetic structure and approximate Bayesian computation analyses reveal the southern origin and northward dispersal of the oriental fruit moth *Grapholita molesta* (Lepidoptera: Tortricidae) in its native range. Mol. Ecol..

[9] Takano S (2017). Unique clade of alphaproteobacterial endosymbionts induces complete cytoplasmic incompatibility in the coconut beetle. Proc. Natl. Acad. Sci. USA.

[10] Radcliffe EB, Flanders KL (1998). Biological control of alfalfa weevil in North America. Integr. Pest Manag. Rev..

[11] Kuwata R, Tokuda M, Yamaguchi D, Yukawa J (2005). Coexistence of two mitochondrial DNA haplotypes in Japanese populations of Hypera postica (Col., Curculionidae). J. Appl. Entomol..

[12] Skuhrovec J (2005). Host plants of weevils of the genus *Hypera* (Coleoptera: Curculionidae) occurring in the Czech Republic. Klapalekiana.

[13] Wood, K. A., Armbrust, E. J., Bartell, D. P. & Irwin, B. J. The literature of arthropods associated with alfalfa. V. A bibliography of the alfalfa weevil, *Hypera postica* (Gyllenhal), and the Egyptian alfalfa weevil, *Hypera brunneipennis* (Boheman) (Coleoptera: Curculionidae). Illinois Agricultural Experimental Station, Special Publication, 54 (1978).

[14] Kimura, H., Okumura, M. & Yoshida, T. Emergence of and recent damage by the alfalfa weevil. Shokubutsu Boeki (Plant Protection*)* 42, 498–501 (in Japanese) (1988).

[15] CAB International crop protection compendium. CAB International. http://www.cabicompendium.org/cpc/home.asp (2013).

[16] Titus EG (1910). On the life history of the alfalfa leaf-weevil. J. Econ. Entomol..

[17] Wehrle LP (1940). The discovery of an alfalfa weevil (*Hypera brunneipennis* Boheman) in Arizona. J. Econ. Entomol..

[18] Poos FW, Bissell TL (1953). The alfalfa weevil in Maryland. J. Econ. Entomol..

[19] Volker KC, Simpson RG (1975). Behavior of alfalfa weevil larvae affecting the establishment of *Tetrastichus incertus* in Colorado. Environ. Entomol..

[20] Salt G, van den Bosch R (1967). The defense reactions of three species of *Hypera* (Coleoptera, Curculionidae) to an Ichneumon wasp. J. Invertebr. Pathol..

[21] Maund CM, Hsiao TH (1991). Differential encapsulation of two *Bathyplectes* parasitoids among alfalfa weevil strains, *Hypera postica* (Gyllenhal). Can. Entomol..

[22] Hsiao, T. H. Studies of interactions between alfalfa weevil strains, *Wolbachia* endosymbionts and parasitoids. In The ecology of agricultural pests: biochemical approaches (eds, Symondson, W. O. C. & Liddell, J. E.). 57–71 (Chapman & Hall, 1996).

[23] Hsiao TH, Stutz JM (1985). Discrimination of alfalfa weevil strains by allozyme analysis. Entomol. Exp. Appl..

[24] Erney SJ, Pruess KP, Danielson SD, Powers TO (1996). Molecular differentiation of alfalfa weevil strains (Coleoptera: Curculionidae). Ann. Entomol. Soc. Am..

[25] Böttger JAA (2013). Phylogenetic analysis of the alfalfa weevil complex (Coleoptera: Curculionidae) in North America. J. Econ. Entomol..

[26] Iwase S, Nakahira K, Tuda M, Kagoshima K, Takagi M (2015). Host-plant dependent population genetics of the invading weevil *Hypera postica*. Bull. Entomol. Res..

[27] White CE, Armbrust EJ, Ashley J (1972). Cross-mating studies of eastern and western strains of alfalfa weevil. J. Econ. Entomol..

[28] Iwase S, Tani S (2016). New haplotype and inter-strain reproductive compatibility of *Wolbachia*-uninfected alfalfa weevil, *Hypera postica* (Coleoptera: Curculionidae), in Japan. Entomol. Sci..

[29] Werren JH (1997). Biology of *Wolbachia*. Annu. Rev. Entomol..

[30] LePage DP (2017). Prophage WO genes recapitulate and enhance *Wolbachia*-induced cytoplasmic incompatibility. Nature.

[31] Bailly-Bechet M (2017). How long does *Wolbachia* remain on board?. Mol. Biol. Evol..

[32] Hale LR, Hoffmann AA (1990). Mitochondrial DNA polymorphism and cytoplasmic incompatibility in natural populations of *Drosophila simulans*. Evolution.

[33] Ballard JWO, Kreitman M (1994). Unravelling selection in the mitochondrial genome of *Drosophila*. Genetics.

[34] Johnstone RA, Hurst GDD (1996). Maternally inherited male-killing microorganisms may confound interpretation of mitochondrial DNA variability. Biol. J. Linn. Soc..

[35] Jiggins FM (2003). Male-killing *Wolbachia* and mitochondrial DNA: selective sweeps, hybrid introgression and parasite population dynamics. Genetics.

[36] Werren JH, Baldo L, Clark ME (2008). *Wolbachia*: master manipulators of invertebrate biology. Nat. Rev. Microbiol..

[37] Shoemaker DD, Dyer KA, Ahrens M, McAbee K, Jaenike J (2004). Decreased diversity but increased substitution rate in host mtDNA as a consequence of *Wolbachia* endosymbiont infection. Genetics.

[38] Cariou M, Duret L, Charlat S (2017). The global impact of *Wolbachia* on mitochondrial diversity and evolution. J. Evol. Biol..

[39] Teixeira L, Ferreira A, Ashburner M (2008). The bacterial symbiont *Wolbachia* induces resistance to RNA viral infections in *Drosophila melanogaster*. PLoS Biol..

[40] Brownlie JC (2009). Evidence for metabolic provisioning by a common invertebrate endosymbiont, *Wolbachia pipientis*, during periods of nutritional stress. PLoS Pathog..

[41] Rand DM, Haney RA, Fry AJ (2004). Cytonuclear coevolution: the genomics of cooperation. TRENDS Ecol. Evol..

[42] Arnqvist G (2010). The genetic architecture of metabolic rate: environment specific epistasis between mitochondrial and nuclear genes in an insect. Evolution.

[43] Blickenstaff CC (1965). Partial intersterility of eastern and western US strains of the alfalfa weevil. Ann. Entomol. Soc. Am..

[44] Hsiao TH, Hsiao C (1985). Hybridization and cytoplasmic incompatibility among alfalfa weevil strains. Entomol. Exp. Appl..

[45] Laven H (1967). Eradication of *Culex pipiens fatigans* through cytoplasmic incompatibility. Nature.

[46] Iwase S (2015). Dynamics of infection with *Wolbachia* in *Hypera postica* (Coleoptera: Curculionidae) during invasion and establishment. Biol. Invasions.

[47] Sanaei E (2019). Global genetic diversity, lineage distribution and *Wolbachia* infection of the alfalfa weevil *Hypera postica* (Coleoptera: Curculionidae). Ecol. Evol..

[48] Ros VID, Fleming VM, Feil EJ, Breeuwer JAJ (2009). How diverse is the genus *Wolbachia*? Multiple-gene sequencing reveals a putatively new *Wolbachia* supergroup recovered from spider mites (Acari: Tetranychidae). Appl. Environ. Microbiol..

[49] Avise JC (2000). Phylogeography: The history and formation of species.

[50] Narita S, Nomura M, Kato Y, Fukatsu T (2006). Genetic structure of sibling butterfly species affected by *Wolbachia* infection sweep: evolutionary and biogeographical implications. Mol. Ecol..

[51] Raychoudhury R (2010). Phylogeography of *Nasonia vitripennis* (Hymenoptera) indicates a mitochondrial–*Wolbachia* sweep in North America. Heredity.

[52] Jäckel R, Mora D, Dobler S (2013). Evidence for selective sweeps by *Wolbachia* infections: phylogeny of *Altica* leaf beetles and their reproductive parasites. Mol. Ecol..

[53] Jiang W (2014). *Wolbachia* infection status and genetic structure in natural populations of *Polytremis nascens* (Lepidoptera: Hesperiidae). Infect. Genet. Evol..

[54] Jansen VAA, Turelli M, Godfray HCJ (2008). Stochastic spread of *Wolbachia*. Proc. R. Soc. Lond. B Biol. Sci..

[55] Clancy DJ, Hoffmann AA (1998). Environmental effects on cytoplasmic incompatibility and bacterial load in *Wolbachia*-infected *Drosophila simulans*. Entomol. Exp. Appl..

[56] Bordenstein SR, Bordenstein SR (2011). Temperature affects the tripartite interactions between bacteriophage WO, *Wolbachia*, and cytoplasmic incompatibility. PLoS ONE.

[57] Kamo T (2008). Limited distribution of natural cyanamide in higher plants: Occurrence in *Vicia villosa* subsp varia, *V. cracca*, and *Robinia pseudo-acacia*. Phytochemistry.

[58] Megías C, Cortes-Giraldo I, Giron-Calle J, Alaiz M, Vioque J (2016). Free amino acids, including canavanine, in the seeds from 32 *Vicia* species belonging to subgenus *Vicilla*. Biocatal. Agric. Biotechnol..

[59] Rosenthal GA, Dahlman DL (1991). Incorporation of L-canavanine into proteins and the expression of its antimetabolic effects. J. Agric. Food Chem..

[60] Kamo, T., Tokuoka. Y. & Miyazaki, M. Quantification of canavanine, 2-aminoethanol, and cyanamide in *Aphis craccivora* and its host plants, *Robinia pseudoacacia* and *Vicia angustifolia*: Effects of these compounds on larval survivorship of *Harmonia axyridis*. J. Chem. Ecol. **38**, 1552–1560 (2012). 10.1007/s10886-012-0220-9 23179101

[61] Hewitt GM (1999). Post-glacial re-colonization of European biota. Biol. J. Linn. Soc..

[62] Schmitt T (2007). Molecular biogeography of Europe: Pleistocene cycles and postglacial trends. Front. Zool..

[63] Taberlet P, Fumagalli L, Wust-Saucy AG, Cosson JF (1998). Comparative phylogeography and postglacial colonization routes in Europe. Mol. Ecol..

[64] Jordal BH, Kambestad M (2013). DNA barcoding of bark and ambrosia beetles reveals excessive NUMTs and consistent east-west divergence across Palearctic forests. Mol. Ecol. Resour..

[65] Quiros, C. F. & Bauchan, G. R. The genus *Medicago* and the origin of the *Medicago sativa* complex. In Alfalfa and alfalfa improvement (eds, Hanson, A. A., Barnes, D. K. & Hill, R. R.). 93–124 (American Society of Agronomy, Crop Science Society of America, Soil Science Society of America, 1988).

[66] Small E (2011). Alfalfa and Relatives: Evolution and Classification of *Medicago*.

[67] FAO Statistics Division. FAOSTAT: Crops and livestock products. http://www.fao.org/faostat/en/#data/TP (2017).

[68] Simon CA (1994). Evolution, weighting, and phylogenetic utility of mitochondrial gene sequences and a compilation of conserved polymerase chain reaction primers. Ann. Entomol. Soc. Am..

[69] Kim CG (2000). Pattern of morphological diversification in the *Leptocarabus* ground beetles (Coleoptera: Carabidae) as deduced from mitochondrial ND5 gene and nuclear 28S rDNA sequences. Mol. Biol. Evol..

[70] Holden PR, Brookfield JFY, Jones P (1993). Cloning and characterization of an ftsZ homologue from a bacterial symbiont of *Drosophila melanogaster*. Mol. Gen. Genet..

[71] Kondo NI (2011). *Wolbachia* infections in world populations of bean beetles (Coleoptera: Chrysomelidae: Bruchinae) infesting cultivated and wild legumes. Zool. Sci..

[72] Baldo L (2006). Multilocus sequence typing system for the endosymbiont *Wolbachia pipientis*. Appl. Environ. Microbiol..

[73] Huelsenbeck JP, Ronquist F (2001). MrBayes: Bayesian inference of phylogeny. Biometrics.

[74] Nylander, J. A. A. MrAIC.pl. Program distributed by the author. Uppsala: Evolutionary Biology Centre, Uppsala University (2004).

[75] Rambaut, A. & Drummond, A. J. Tracer v1.5, http://beast.bio.ed.ac.uk/ (2009).

[76] Tajima F (1989). Statistical methods to test for nucleotide mutation hypothesis by DNA polymorphism. Genetics.

[77] Fu Y-X, Li W-H (1993). Statistical tests of neutrality of mutations. Genetics.

[78] Librado P, Rozas J (2009). DnaSP v5: A software for comprehensive analysis of DNA polymorphism data. Bioinformatics.

[79] Yang Z (2007). PAML 4: A program package for phylogenetic analysis by maximum likelihood. Mol. Biol. Evol..

[80] Song H, Buhay JE, Whiting MF, Crandall KA (2008). Many species in one: DNA barcoding overestimates the number of species when nuclear mitochondrial pseudogenes are coamplified. Proc. Natl. Acad. Sci. USA.

[81] Haran J, Koutroumpa F, Magnoux E, Roques A, Roux G (2015). Ghost mtDNA haplotypes generated by fortuitous NUMTs can deeply disturb infra-specific genetic diversity and phylogeographic pattern. J. Zoolog. Syst. Evol. Res..

[82] Clement M, Snell Q, Walker P, Posada D, Crandall K (2002). TCS: Estimating gene genealogies. Parallel Distrib. Proces. Symp. Int. Proc..

[83] Tajima F (1983). Evolutionary relationship of DNA sequences in finite populations. Genetics.

[84] Excoffier L, Lischer HEL (2010). Arlequin suite ver 3.5: A new series of programs to perform population genetics analyses under Linux and Windows. Mol. Ecol. Resour..

[85] Wright S (1943). Isolation by distance. Genetics.

[86] Mantel N (1967). The detection of disease clustering and a generalized regression approach. Cancer Res..

[87] Raymond M, Rousset F (1995). Genepop (version 1.2): Population genetics software for exact tests and ecumenicism. J. Hered..

[88] Nei M, Li WH (1979). Mathematical model for studying genetic variation in terms of restriction endonucleases. Proc. Natl. Acad. Sci. USA.

[89] Lemey P, Rambaut A, Drummond AJ, Suchard MA (2009). Bayesian phylogeography finds its roots. PLoS Comput. Biol..

[90] Suchard MA (2018). Bayesian phylogenetic and phylodynamic data integration using BEAST 1.10. Virus Evol..

[91] Drummond AJ, Suchard MA, Xie D, Rambaut A (2012). Bayesian phylogenetics with BEAUti and the BEAST 1.7. Mol. Biol. Evol..

[92] Bielejec F (2016). SpreaD3: Interactive visualization of spatiotemporal history and trait evolutionary processes. Mol. Biol. Evol..

