# Supplementary material for: Diversification, selective sweep, and body size in the invasive Palearctic alfalfa weevil infected with Wolbachia
Source: Sci Rep. 2021 May 6;11:9664. doi: 10.1038/s41598-021-88770-y (PMC8102540; doi:10.1038/s41598-021-88770-y)
Supplement: Supplementary file 1 — Supplementary Information. [file 41598_2021_88770_MOESM1_ESM.docx]

Diversification, selective sweep, and body size in the invasive Palearctic alfalfa weevil infected with *Wolbachia*

Midori Tuda^1,2*^, Shun-ichiro Iwase^1*^, Khadim Kébé ^3*^, Julien Haran^4*^, Jiri Skuhrovec^5*^, Ehsan Sanaei^6^, Naomichi Tsuji^7^, Attila Podlussány^8^, Ottó Merkl^8^, Ahmed H. El-Heneidy^9^, Katsura Morimoto^10^

^1^Institute of Biological Control, Faculty of Agriculture, Kyushu University, Fukuoka, Japan

^2^Laboratory of Insect Natural Enemies, Department of Bioresource Sciences, Faculty of Agriculture, Kyushu University, Fukuoka, Japan

^3^GRBA-BE, LE3PI Laboratory, Department of Chemical Engineering and Applied Biology, Polytechnic Higher School of Dakar, Senegal

^4^CBGP, Cirad, Montpellier SupAgro, INRA, IRD, Univ. Montpellier, Montpellier, France

^5^Group Function of Invertebrate and Plant Biodiversity in Agro-ecosystems, Crop Research Institute, Drnovska, Praha, Czech Republic

^6^School of Biological Sciences, University of Queensland, Brisbane, Australia

^7^Entomological Laboratory, Graduate School of Bioresource and Bioenvironmental Sciences, Kyushu University, Fukuoka, Japan

^8^Hungarian Natural History Museum, Budapest, Hungary

^9^Department of Biological Control, Plant Protection Research Institute, Agricultural Research Center, Giza, Egypt

^10^Nata-danchi, Fukuoka, Japan

**Supplementary Table S1.** Mitochondrial genetic distance between populations. Diagonal elements are the average number of pairwise differences within population (π_X_); values below the diagonal are the corrected average pairwise differences (π_XY_ − (π_X_ + π_Y_)/2). Bold values have *p* < 0.01.

|  | Prague | Amsterdam | Nida Basin | Daugavpils | Budapest | Adyliget | Chaussy | Orleans | Aurade | Combaillaux | Saint-Paul-sur-Ubaye | Casamozza | La Canada | Maremma | Puglia | Sparti | Crucea |
| --- | --- | --- | --- | --- | --- | --- | --- | --- | --- | --- | --- | --- | --- | --- | --- | --- | --- |
| Prague | 0.00 |  |  |  |  |  |  |  |  |  |  |  |  |  |  |  |  |
| Amsterdam | 0.00 | 0.00 |  |  |  |  |  |  |  |  |  |  |  |  |  |  |  |
| Nida Basin | 0.00 | 0.00 | 2.00 |  |  |  |  |  |  |  |  |  |  |  |  |  |  |
| Daugavpils | 1.00 | 1.00 | 1.00 | 0.00 |  |  |  |  |  |  |  |  |  |  |  |  |  |
| Budapest | 0.00 | 0.00 | 0.00 | 1.00 | 0.00 |  |  |  |  |  |  |  |  |  |  |  |  |
| Adyliget | 0.00 | 0.00 | 0.00 | 1.00 | 0.00 | 0.44 |  |  |  |  |  |  |  |  |  |  |  |
| Chaussy | 0.00 | 0.00 | 0.00 | 1.00 | 0.00 | 0.00 | 0.00 |  |  |  |  |  |  |  |  |  |  |
| Orleans | 0.00 | 0.00 | 0.00 | 0.83 | 0.00 | 0.00 | 0.00 | 6.50 |  |  |  |  |  |  |  |  |  |
| Aurade | 0.00 | 0.00 | 0.00 | 1.00 | 0.00 | 0.00 | 0.00 | 0.00 | 0.67 |  |  |  |  |  |  |  |  |
| Combaillaux | 2.14 | 2.14 | 2.14 | 3.14 | 2.14 | **2.11** | 2.14 | **1.62** | 2.14 | 7.72 |  |  |  |  |  |  |  |
| Saint-Paul-sur-Ubaye | 39.00 | 39.00 | 39.00 | 38.00 | 39.00 | 39.00 | 39.00 | 32.50 | 39.00 | 34.92 | 1.00 |  |  |  |  |  |  |
| Casamozza | **38.55** | 38.55 | 38.55 | 37.55 | **38.55** | **38.55** | **38.55** | **32.18** | 38.55 | **34.73** | 0.45 | 4.90 |  |  |  |  |  |
| La Canada | **25.37** | 25.37 | 25.37 | 24.70 | 25.37 | **25.37** | **25.37** | **20.06** | **25.37** | **22.43** | 0.03 | 0.32 | 17.60 |  |  |  |  |
| Maremma | 33.33 | 33.33 | 32.33 | 32.33 | 33.33 | **33.33** | 33.33 | **28.03** | 33.33 | **30.66** | 8.67 | 8.48 | 4.53 | 12.00 |  |  |  |
| Puglia | 41.00 | 41.00 | 41.00 | 40.00 | 41.00 | 41.00 | 41.00 | 34.92 | 41.00 | 37.58 | 6.50 | 1.95 | 5.53 | 12.67 | 2.00 |  |  |
| Sparti | **28.45** | 28.45 | 28.45 | 27.45 | **28.45** | **28.45** | **28.45** | **23.17** | 28.45 | **25.12** | 3.85 | 1.92 | 0.78 | 4.65 | 2.65 | 15.50 |  |
| Crucea | **34.25** | 34.25 | 34.25 | 33.25 | **34.25** | **34.25** | **34.25** | **28.25** | 34.25 | **30.79** | 0.95 | −0.32 | −0.28 | 4.18 | 1.85 | −0.58 | 10.70 |
| Knezha | **26.64** | 26.64 | 26.64 | 27.04 | **26.64** | **26.64** | **26.64** | **21.79** | **26.64** | **22.29** | 7.44 | **7.79** | 3.74 | **8.31** | 11.74 | 4.89 | 4.89 |
| Lozitsa | **21.98** | 21.98 | 21.98 | 22.98 | **21.98** | **21.98** | **21.98** | **18.27** | **21.98** | **19.65** | 16.48 | **17.03** | **10.43** | **15.23** | 20.98 | **13.31** | **13.63** |
| Zagreb | 0.00 | 0.00 | 0.00 | 1.00 | 0.00 | 0.00 | 0.00 | 0.00 | 0.00 | 2.14 | 39.00 | 38.55 | 25.20 | 32.67 | 41.00 | 28.45 | 34.05 |
| Ouzoud Fall | **37.79** | 37.79 | 37.79 | 36.79 | **37.79** | **37.79** | **37.79** | **31.45** | **37.79** | **33.97** | −0.01 | −0.24 | −0.38 | **7.16** | 4.39 | 2.32 | 0.02 |
| Ouarzazate | 40.00 | 40.00 | 40.00 | 39.00 | 40.00 | 40.00 | 40.00 | **33.92** | 40.00 | 36.58 | 5.75 | 2.25 | 4.62 | 11.33 | 1.00 | 1.85 | 1.15 |
| Benghazi | **34.60** | 34.60 | 34.60 | 33.60 | **34.60** | **34.60** | **34.60** | **28.57** | 34.60 | **30.65** | 0.40 | 0.75 | −0.63 | 7.33 | 5.60 | 1.13 | −0.03 |
| Sakha | 33.00 | 33.00 | 33.00 | 32.00 | 33.00 | **33.00** | 33.00 | **27.00** | 33.00 | **29.51** | 0.00 | 0.88 | −0.86 | 2.89 | 6.33 | 1.32 | −0.62 |
| Gaash | 28.00 | 28.00 | 28.00 | 27.00 | 28.00 | 28.00 | 28.00 | 22.42 | 28.00 | 24.81 | 0.00 | 0.75 | −1.97 | 0.67 | 6.00 | 0.15 | −1.35 |
| Adana | 38.33 | 38.33 | 38.33 | 37.33 | 38.33 | **38.33** | 38.33 | **32.25** | 38.33 | **34.92** | 3.83 | **2.78** | 1.78 | 4.83 | 6.83 | 1.88 | 1.48 |
| Metsamor | 36.00 | 36.00 | 36.00 | 35.00 | 36.00 | 36.00 | 36.00 | 29.83 | 36.00 | 32.25 | 0.00 | −0.45 | −1.30 | 5.00 | 4.50 | 0.65 | −0.55 |
| Azerbaijan | 37.50 | 37.50 | 37.50 | 36.50 | 37.50 | 37.50 | 37.50 | **31.71** | 37.50 | 34.08 | 7.00 | 5.35 | 3.95 | 4.67 | 7.50 | 1.85 | 2.25 |
| Taleghan | **24.52** | 24.52 | 24.52 | 24.77 | **24.52** | **24.52** | **24.52** | **19.97** | **24.52** | **20.99** | 8.89 | **9.24** | **4.43** | **8.18** | 13.27 | 5.99 | **6.12** |
| Hamedan | 37.33 | 37.33 | 37.33 | 36.33 | 37.33 | **37.33** | 37.33 | **31.28** | 37.33 | **33.92** | 2.67 | 1.55 | 0.59 | 4.56 | 5.67 | 1.52 | 0.85 |
| Dasht-E-Arzhan | 40.00 | 40.00 | 40.00 | 39.00 | 40.00 | 40.00 | 40.00 | 34.00 | 40.00 | 36.58 | 6.50 | 3.95 | 4.87 | 9.33 | 4.50 | 3.75 | 2.05 |
| Anau | 24.00 | 24.00 | 24.00 | 23.00 | 24.00 | 24.00 | 24.00 | 19.58 | 24.00 | 21.58 | 10.00 | 10.35 | 5.03 | 6.17 | 14.00 | 5.65 | 6.25 |
| Jangy-Talap | **37.83** | 37.83 | 37.83 | 36.83 | 37.83 | **37.83** | 37.83 | **32.00** | 37.83 | **34.42** | 6.83 | **4.98** | 4.12 | 6.00 | 7.33 | **2.68** | 3.08 |

|  | Knezha | Lozitsa | Zagreb | Ouzoud Falls | Ouarzazate | Benghazi | Sakha | Gaash | Adana | Metsamor | Azerbaijan | Taleghan | Hamedan | Dasht-E-Arzhan | Anau | Jangy-Talap |
| --- | --- | --- | --- | --- | --- | --- | --- | --- | --- | --- | --- | --- | --- | --- | --- | --- |
| Knezha | 13.11 |  |  |  |  |  |  |  |  |  |  |  |  |  |  |  |
| Lozitsa | 3.28 | 14.79 |  |  |  |  |  |  |  |  |  |  |  |  |  |  |
| Zagreb | 26.64 | 21.98 | 1.00 |  |  |  |  |  |  |  |  |  |  |  |  |  |
| Ouzoud Falls | **7.07** | **16.05** | 37.79 | 5.22 |  |  |  |  |  |  |  |  |  |  |  |  |
| Ouarzazate | 9.44 | 18.98 | 40.00 | **4.09** | 5.00 |  |  |  |  |  |  |  |  |  |  |  |
| Benghazi | **5.72** | **14.63** | 34.60 | 0.17 | 4.00 | 6.80 |  |  |  |  |  |  |  |  |  |  |
| Sakha | 4.64 | **12.65** | 33.00 | 0.09 | 5.33 | 0.40 | 7.33 |  |  |  |  |  |  |  |  |  |
| Gaash | 3.34 | 10.73 | 28.00 | −0.21 | 5.00 | −0.20 | −3.33 | 12.00 |  |  |  |  |  |  |  |  |
| Adana | 7.38 | **16.50** | 38.33 | **2.17** | 5.08 | 2.73 | 2.00 | 0.83 | 3.33 |  |  |  |  |  |  |  |
| Metsamor | 5.74 | 14.86 | 36.00 | −0.91 | 3.50 | −0.60 | −0.33 | −1.00 | −0.67 | 7.00 |  |  |  |  |  |  |
| Azerbaijan | 7.44 | 16.61 | 36.50 | 5.09 | 5.00 | 4.80 | 4.00 | 2.50 | 1.33 | 2.00 | 7.00 |  |  |  |  |  |
| Taleghan | −0.78 | 2.27 | 24.52 | **8.39** | 11.14 | **7.17** | 5.43 | 3.83 | **8.48** | 7.02 | 8.83 | 13.96 |  |  |  |  |
| Hamedan | 6.91 | **15.57** | 37.33 | 1.09 | 4.50 | 1.40 | 1.44 | 0.33 | −0.33 | −1.67 | 2.00 | 7.81 | 5.33 |  |  |  |
| Dasht-E-Arzhan | **10.84** | 19.86 | 40.00 | 4.09 | 5.00 | 4.80 | 4.67 | 3.50 | 4.83 | 4.00 | 6.50 | 11.64 | 4.67 | 4.00 |  |  |
| Anau | 1.94 | 5.23 | 24.00 | 9.29 | 12.00 | 7.80 | 5.33 | 3.00 | 8.33 | 7.50 | 8.75 | 0.77 | 7.67 | 12.00 | 10.00 |  |
| Jangy-Talap | **8.23** | **17.53** | 37.33 | **5.07** | 5.33 | 5.53 | 4.33 | 2.83 | 1.67 | 2.08 | −0.17 | **9.63** | 2.17 | 8.33 | 9.58 | 7.33 |

**Supplementary Table S2.** Nuclear genetic distance between populations. Diagonal elements are the average number of pairwise differences within population (π_X_); values below the diagonal are the corrected average pairwise differences (π_XY_ − (π_X_ + π_Y_)/2). Bold values have *p* < 0.01.

|  | Prague | Amsterdam | Budapest | Adyliget | Chaussy | Orleans | Aurade | Saint-Paul-sur-Ubaye | Casamozza | LaCanada | Puglia | Knezha | Ouzoud Falls | Sakha | Gaash |
| --- | --- | --- | --- | --- | --- | --- | --- | --- | --- | --- | --- | --- | --- | --- | --- |
| Prague | 3.00 |  |  |  |  |  |  |  |  |  |  |  |  |  |  |
| Amsterdam | 2.70 | 0.00 |  |  |  |  |  |  |  |  |  |  |  |  |  |
| Budapest | 0.10 | 1.5 | 3.00 |  |  |  |  |  |  |  |  |  |  |  |  |
| Adyliget | 0.56 | 2.1 | -0.30 | 8.20 |  |  |  |  |  |  |  |  |  |  |  |
| Chaussy | -0.43 | 2.42 | -0.58 | 0.27 | 3.17 |  |  |  |  |  |  |  |  |  |  |
| Orleans | 0.30 | 1.36 | 0.31 | 0.48 | 0.50 | 3.29 |  |  |  |  |  |  |  |  |  |
| Aurade | 0.50 | 1.00 | 0.17 | 0.30 | 0.58 | -0.44 | 4.00 |  |  |  |  |  |  |  |  |
| Saint-Paul-sur-Ubaye | 14.50 | 14.00 | 13.00 | 13.50 | 13.67 | 14.46 | 14.33 | 0.00 |  |  |  |  |  |  |  |
| Casamozza | -0.10 | 1.33 | -0.17 | 0.03 | -0.25 | -0.24 | -0.11 | 13.33 | 5.33 |  |  |  |  |  |  |
| LaCanada | 1.33 | 1.87 | 0.95 | **1.67** | 0.83 | 1.42 | 1.42 | 13.53 | -0.30 | 3.60 |  |  |  |  |  |
| Puglia | 0.90 | 3.00 | 1.00 | 0.90 | 1.17 | **0.36** | 0.00 | 16.00 | 1.00 | 3.37 | 0.00 |  |  |  |  |
| Knezha | 4.50 | 7.00 | 4.00 | 4.90 | 4.17 | **5.36** | 4.67 | 16.00 | 4.33 | 5.37 | 6.00 | 0.00 |  |  |  |
| Ouzoud Falls | **2.89** | 2.29 | 2.29 | **3.01** | **2.30** | **2.32** | **2.20** | 14.04 | 0.33 | 0.05 | 4.66 | 6.54 | 3.93 |  |  |
| Sakha | 0.50 | 2.33 | 0.00 | -0.17 | 0.33 | 0.69 | 0.67 | 14.00 | -0.11 | 1.14 | 1.33 | 4.67 | **2.83** | 5.33 |  |
| Gaash | -0.30 | 1.00 | -0.75 | -0.40 | -0.46 | -0.64 | -1.00 | 13.50 | -1.00 | -0.05 | 0.00 | 4.00 | 0.97 | -0.33 | 6.00 |
| Metsamor | 3.50 | 4.00 | 3.00 | 3.90 | 3.17 | **3.36** | 3.00 | 15.00 | 2.00 | 2.03 | 5.00 | 7.00 | 2.04 | 3.67 | 2.00 |
| Taleghan | 4.50 | 3.00 | 3.50 | 4.30 | 3.92 | **3.76** | 3.33 | 15.00 | 2.33 | 1.53 | 6.00 | 8.00 | 1.54 | 4.00 | 2.00 |
| Hamedan | -0.40 | 0.00 | -0.75 | -0.60 | -0.46 | -1.04 | -1.33 | 12.50 | -1.50 | -0.72 | 0.00 | 4.00 | 0.10 | -1.00 | -2.00 |
| Jangy-Talap | 1.83 | 3.00 | 0.00 | 1.17 | 0.83 | **2.29** | 2.22 | 13.33 | 1.00 | 1.81 | 3.33 | 5.33 | **2.87** | 0.89 | 1.00 |

|  | Metsamor | Taleghan | Hamedan | Jangy-Talap |
| --- | --- | --- | --- | --- |
| Metsamor | 0.00 |  |  |  |
| Taleghan | 3.00 | 0.00 |  |  |
| Hamedan | 1.00 | 1.00 | 7.00 |  |
| Jangy-Talap | 3.67 | 4.00 | 0.67 | 4.00 |

**Supplementary Table S3.** Host, isolate/strain and supergroup of sequences used in the phylogenetic reconstruction of *Wolbachia.*

| Supergroup | Host | Host order | Isolate/strain | Accession |
| --- | --- | --- | --- | --- |
| A | *Nasonia longicornis* | Hymenoptera | 16 | DQ842331 (ftsZ), DQ842295 (coxA), DQ842406 (hcpA) |
| A | *Drosophila melanogaster* | Diptera | wMel | AE017196 (ftsZ, coxA, hcpA) |
| B | *Encarsia formosa* | Hymenoptera | 33 | DQ842324 (ftsZ), DQ842288 (coxA), DQ842399 (hcpA) |
| B | *Nasonia vitripennis* | Hymenoptera | 34 | DQ842333 (ftsZ), DQ842297 (coxA), DQ842408 (hcpA) |
| B | *Culex pipientis* | Diptera | wPip | AM99887 (ftsZ, coxA, hcpA) |
| B | *Tetranychus urticae* | Acari: Trombidiformes | ST279 | JX094400 (ftsZ), JX094413 (coxA), JX094405 (hcpA) |
| B | *Nilaparvata muiri* | Hemiptera | Jinhua clone 1 | HQ404753 (ftsZ), HQ404751 (coxA), HQ404752 (hcpA) |
| B | *Tribolium confusum* | Coleoptera | 20 | DQ842337 (ftsZ), DQ842301 (coxA), DQ842412 (hcpA) |
| B | *Bryobia praetiosa* | Acari: Trombidiformes | NL12 | EU499322 (ftsZ) |
| B | *Hypera postica* | Coleoptera | wHypera1/Western | MW389117 (ftsZ), MW389113 (coxA), MW389115 (hcpA) |
| B | *Eurema hecabe* | Lepidoptera | wCI/ISG1 | AB592919 (ftsZ), AB592909 (coxA), AB592914 (hcpA) |
| B | *Eurema hecabe* | Lepidoptera | wFem/ISG1 | AB592920 (ftsZ), AB592910 (coxA), AB592915 (hcpA) |
| B | *Bicyclus taenias* | Lepidoptera | wBtae | KY658588 (ftsZ), KY658647 (coxA), KY658625 (hcpA) |
| B | *Hypera postica* | Coleoptera | wHyperaJ/Egyptian | MW389118 (ftsZ), MW389114 (coxA), MW389116 (hcpA) |
| B | *Porcellionides pruinosus* | Isopoda | wPruIII | LK052874 (coxA), LK052877 (hcpA) |
| C | *Dirofilaria immitis* | Nematoda: Rhabditida | wDimm/FR3 | NZ_CP046578 (ftsZ, coxA, hcpA) |
| D | *Brugia malayi* | Nematoda: Spirurida | TRS | CP034333 (ftsZ, coxA, hcpA) |
| E | *Folsomia candida* | Collembola | wFcan | KT799600 (ftsZ), KT799590 (coxA), KT799610 (hcpA) |
| F | *Coptotermes acinaciformis* | Blattodea | - | FJ390319 (ftsZ), FJ390173 (hcpA) |
| H | *Zootermopsis angusticollis* | Isoptera | - | AY764283 (ftsZ), FJ390248 (coxA), FJ390174 (hcpA) |
|  |  |  |  |  |
| Outgroup | *Anaplasma marginale* strain Florida | | | CP001079 (ftsZ, coxA) |
